# Improved personalized survival prediction of patients with diffuse large B-cell Lymphoma using gene expression profiling

**DOI:** 10.1186/s12885-020-07492-y

**Published:** 2020-10-21

**Authors:** Adrián Mosquera Orgueira, José Ángel Díaz Arias, Miguel Cid López, Andrés Peleteiro Raíndo, Beatriz Antelo Rodríguez, Carlos Aliste Santos, Natalia Alonso Vence, Ángeles Bendaña López, Aitor Abuín Blanco, Laura Bao Pérez, Marta Sonia González Pérez, Manuel Mateo Pérez Encinas, Máximo Francisco Fraga Rodríguez, José Luis Bello López

**Affiliations:** 1 Health Research Institute of Santiago de Compostela (IDIS), Santiago de Compostela, Spain; 2 Department of Hematology, SERGAS, Complexo Hospitalario Universitario de Santiago de Compostela (CHUS), Santiago, Spain; 3 Hospital Clínico Universitario de Santiago de Compostela, Servicio de Hematología, planta 1, Avenida da Choupana s/n, 15706 Santiago de Compostela, Spain; 4 University of Santiago de Compostela, Santiago de Compostela, Spain; 5 Department of Pathology, SERGAS, Complexo Hospitalario Universitario de Santiago de Compostela (CHUS), Santiago de Compostela, Spain

**Keywords:** DLBCL, Lymphoma, Survival, Prediction, Transcriptomics

## Abstract

**Background:**

Thirty to forty percent of patients with Diffuse Large B-cell Lymphoma (DLBCL) have an adverse clinical evolution. The increased understanding of DLBCL biology has shed light on the clinical evolution of this pathology, leading to the discovery of prognostic factors based on gene expression data, genomic rearrangements and mutational subgroups. Nevertheless, additional efforts are needed in order to enable survival predictions at the patient level. In this study we investigated new machine learning-based models of survival using transcriptomic and clinical data.

**Methods:**

Gene expression profiling (GEP) of in 2 different publicly available retrospective DLBCL cohorts were analyzed. Cox regression and unsupervised clustering were performed in order to identify probes associated with overall survival on the largest cohort. Random forests were created to model survival using combinations of GEP data, COO classification and clinical information. Cross-validation was used to compare model results in the training set, and Harrel’s concordance index (c-index) was used to assess model’s predictability. Results were validated in an independent test set.

**Results:**

Two hundred thirty-three and sixty-four patients were included in the training and test set, respectively. Initially we derived and validated a 4-gene expression clusterization that was independently associated with lower survival in 20% of patients. This pattern included the following genes: *TNFRSF9*, *BIRC3*, *BCL2L1* and *G3BP2*. Thereafter, we applied machine-learning models to predict survival. A set of 102 genes was highly predictive of disease outcome, outperforming available clinical information and COO classification. The final best model integrated clinical information, COO classification, 4-gene-based clusterization and the expression levels of 50 individual genes (training set c-index, 0.8404, test set c-index, 0.7942).

**Conclusion:**

Our results indicate that DLBCL survival models based on the application of machine learning algorithms to gene expression and clinical data can largely outperform other important prognostic variables such as disease stage and COO. Head-to-head comparisons with other risk stratification models are needed to compare its usefulness.

## Background

Diffuse Large B-cell Lymphoma (DLBCL) is the most frequent type of lymphoma, accounting for 25% of all cases of non-Hodgkin lymphoma (NHL). DLBCL has an estimated incidence in the United States of 6.9 new cases per 100,000 people/year [1]. Despite its aggressivity, 60–70% of patients achieve curation after first-line immunochemotherapy with R-CHOP (*rituximab, cyclophosphamide, doxorubicin, vincristine, prednisone*) [2]. Nevertheless, the remaining 30–40% of cases exhibit relapsed or refractory disease which frequently precludes a dismal prognosis [3].

Improved biological characterization of DLBCL has led to the identification of new disease subtypes with prognostic implications. DLBCL cases with dual rearrangement of *MYC* and *BCL2* and/or *BCL6*, frequently named *“double-hit”* lymphomas, are associated with significantly shorter survival and have been reclassified as a new group of lymphomas by the World Health Organization [4, 5]. Similarly, using gene expression profiling (GEP), DLBCL can be classified in two broad groups by their cell-of-origin (COO) status, namely germinal center B-cell (GCB)-like and activated B-cell (ABC)-like. Those among the latter show an adverse prognosis with respect to the GCB-like DLBCLs [6]. More recently, different groups reported the identification of new DLBCL subgroups based on co-occurrent genomic alterations [7, 8], paving the path towards a more individualized approach to this disease.

In the meantime, the emergence of artificial intelligence has brought new expectations to the field of medicine, particularly for disease diagnosis and prognostication. Classical models such as cox proportional hazard model and the log-rank test assume that patient outcome consists of a linear combination of covariates, and do not provide decision rules for prediction in the real-world [9]. On the contrary, machine learning (ML) is a field of artificial intelligence that performs outcome prediction based on complex interactions between multiple variables. ML makes little assumptions about the relationship between the dependent and independent variables [10]. In ML, a model is trained with examples and not programmed with human-made rules [11]. In the case of survival data, ML needs to take into account the *time to event* and censoring of the data.

ML has been applied to predict survival in different clinical scenarios with encouraging results. The implementation of ML-based survival models is increasingly popular in order to provide patient-centered risk information that can assist both the clinician and the patient. Kim et al. [12] recently published a deep-learning model that uses clinical parameters to predict survival of oral cancer patients with high concordance with reality. Similarly, random forest-based models have been created to predict 30-day mortality of spontaneous intracerebral hemorrhage [13] and overall mortality of patients with acute kidney injury or in renal transplant recipients [14, 15].

In this study, we used gene expression data from DLBCL cases in order to create new models of survival based on retrospective data. Initially, we sought to identify transcripts and gene expression patterns associated with prognosis. Afterwards, we used this information in order to fit a random forest model capable of predicting overall survival with high-concordance. Comparisons with clinical data and COO classification are provided. We believe that our results will facilitate the establishment of individualized survival predictions in DLBCL.

## Methods

### Data origin and normalization

The gene expression database GSE10846 was used for training and the gene expression database GSE23501 was used as an independent test set (Table 1). GSE10846 contains gene expression data from whole-tissue biopsies of 420 patients diagnosed with DLBCL according to World Health Organization (WHO) 2008 criteria [16], of which we selected 233 cases treated with R-CHOP-like regimens in the first line. GSE23501 contains 69 DLBCL whole-tissue biopsies of patients treated with R-CHOP-like regimens as a first line [17]. Both studies used *Affymetrix HG U133 plus 2.0* arrays for gene expression quantification. As the data from GSE23501 depends from British Columbia biobanks and part of the data from GSE10846 also originated from the same location, we used Spearman correlation to rule out duplicate samples. Indeed we detected 4 samples with almost perfect correlation (> 0.99) which we treated as duplicates and were removed from downstream analysis. A case treated with rituximab, doxorubicin, bleomycin, vinblastine and dacarbazine was also discarded, making a final validation set of 64 cases. No other pairs of samples were strongly correlated at the gene expression level (> 0.9). COO classification was originally deposited with gene expression data, and in both cases this classification was inferred exclusively from gene expression data. Log2-transformed expression data for both cohorts were obtained from the *Gene Expression Omnibus* (GEO) database [18]. Rank normalization was applied to the data in order to make the results comparable.

**Table 1:** Patient characteristics

Cohort		GSE10846	GSE23501
**N. of cases**		**233**	**64**
**Sex (% male)**		**57.50**	**71.87**
**Median Age**		**61.0**	**63.5**
**Median follow-up time (years)**		**2.12**	**2.24**
**COO**	**GCB**	**45.90%**	**57.81%**
	**ABC**	**39.90%**	**29.69%**
	**NC**	**14.20%**	**12.50%**

### Clusterization

The *Mclust* algorithm [19] was used in order to detect the 2 most likely clusters of patients according to the expression of each probe (*Mclust* function, parameter G = 2). Briefly, the *Mclust* algorithm determines the most likely set of clusters according to geometric properties (distribution, volume, and shape). An expectation-maximization algorithm is used for maximum likelihood estimation, and the best model is selected according to Bayes information criteria. The association of each of these probe-level clusters with overall survival was calculated using cox regression. Thereafter, those probes whose clusterization was significantly associated with survival (Bonferroni adjusted *p*-value < 0.05) were selected for multivariate clusterization using the same *Mclust* algorithm. Cluster prediction was performed on the test set using parameters estimated in the training cohort, and cox regression was used to verify the association of this clusterization with overall survival. The Shoenfeld’s test was used to assess the proportional hazards assumption.

### Random forest survival analysis

We initially tested the association of each probe with overall survival in the training set using multivariate cox regression. The Schoenfeld’s method was used to assess the proportional hazards assumption. Those probes which violated this assumption (*p*-value < 0.05) were discarded from further analysis.

Random forest survival models were created with the *rfsrc* function implemented in the *randomForestSRC* package in R [20]. We decided to use this type of model because, in contrast with deep networks, random forest can quantify the relative importance of each variable, and thus enable the filtering of low-importance variables for model reduction and performance improvement. Parameter tuning was performed using the *tune.rfscr* function, which optimizes the *mtry* and *nnodes* variables. Random forests were implemented on survival data of the training cohort. Bootstrapping without replacement was performed with the default *by.node* protocol. Continuous rank probability score (CRPS) was calculated as the integrated Brier score divided by time, and represents the average squared distances between the observed survival status and the predicted survival probability at each time point. CRPS is always a number between 0 and 1, being 0 the best possible result. Survival prediction on the test cohort was performed using the *predict.rfsrc* function with default parameters. Harrel’s concordance index (c-index) was used to assess model discriminative power on the bootstrapped training set and on the test set. C-index reflects to what extent a model predicts the order of events (e.g., deaths) in a cohort [21]. C-indexes below 0.5 indicate poor prediction accuracy, c-indexes near 0.5 indicate random guessing and c-indexes of 1 represent perfect predictions.

Variable reduction was performed by iteratively removing those variables with low importance. Variable importance was calculated with the *vimp* function, and we iteratively removed those samples with negative or low weight (importance < 1 × 10^− 4^). The number of random splits to consider for each candidate splitting variable (“nsplit”) was optimized by testing the performance of the algorithm in the training set with values in the range of 1 to 50 splits. Finally, we chose the best model in terms of c-index for replication in the validation set.

## Results

### Gene expression-based clusterization

Single probe clusterization revealed the existence of four probes strongly associated with overall survival (Bonferroni *p*-value < 0.05). These probes corresponded to the following genes: *TNFRSF9*, *BIRC3*, *BCL2L1* and *G3BP2*. Two of these genes were significantly associated with survival in the test set, namely *TNFRSF9* (*p*-value 0.04) and *BCL2L1* (*p*-value 8.59 × 10^− 3^).

**Fig. 1:**
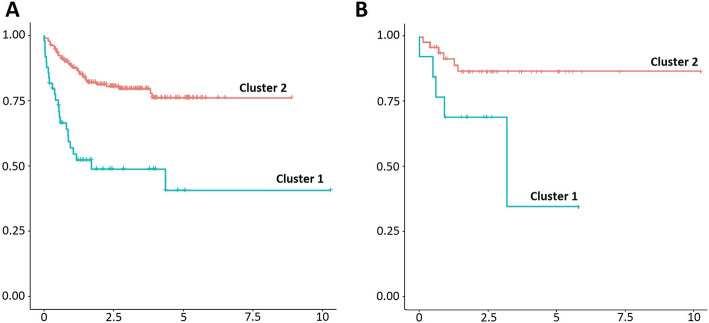
Kaplan-Meier plots of both 4-gene expression based clusters in the training (**a**) and test (**b**) cohorts. The blue line represents patients in the high-risk cluster (cluster 1), and the red line represents the remaining group of patients (cluster 2). Survival probability is represented in the y axis. Time scale (in years) is represented in the x axis

**Fig. 2:**
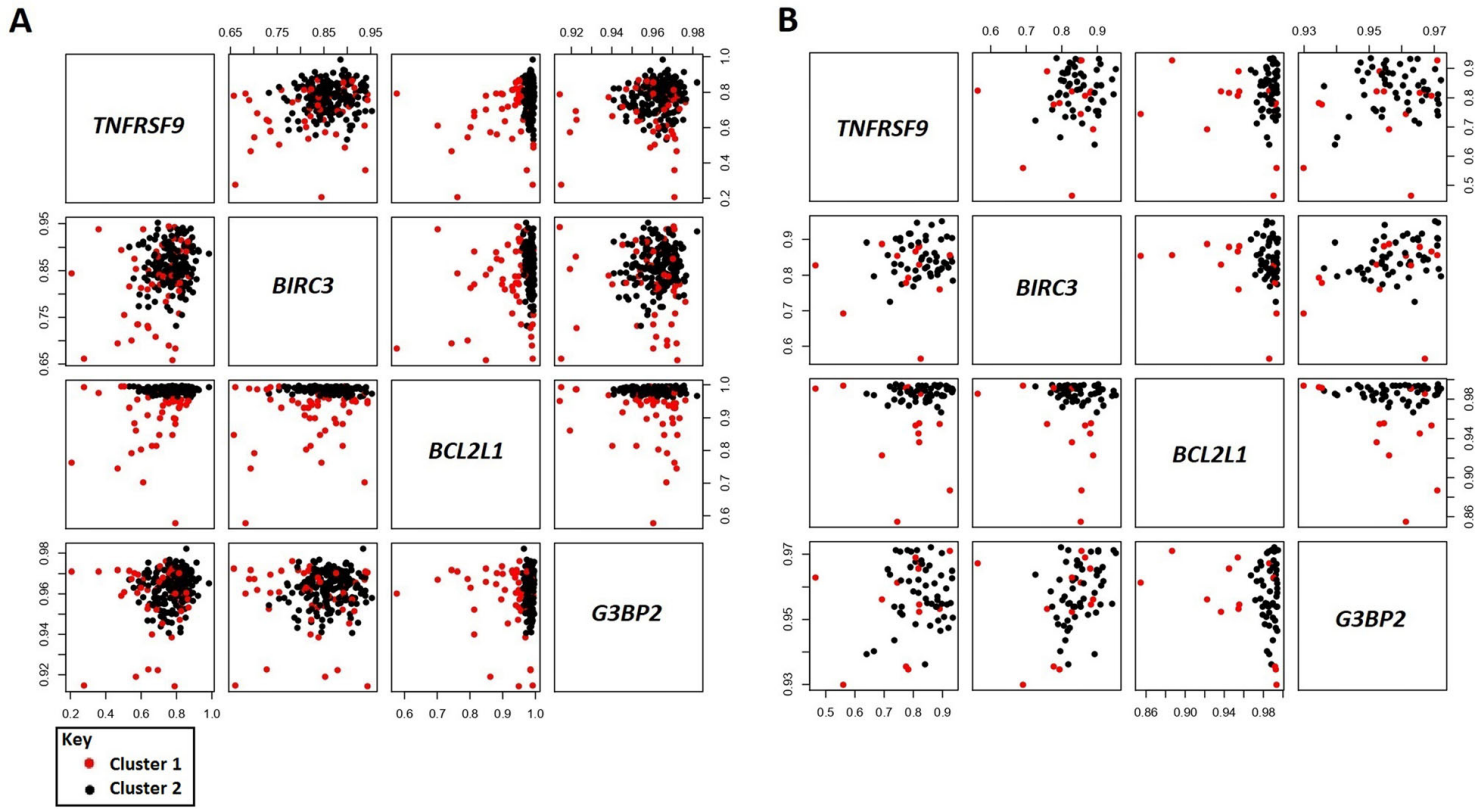
Scatterplot matrix representing the distribution of patients according to the expression of *TNFRSF9*, *BIRC3*, *BCL2L1* and *G3BP2*. Separate plots are provided for the training (**a**) and test (**b**) cohorts. Red dots represent patients in the high-risk cluster (cluster 1), whereas black dots represent the remaining patients (cluster 2)

Multivariate clusterization using the 4 genes identified a cluster of 21.46% of patients with a significantly worse surivival (p-value 1.95 × 10^− 6^, Hazard Ratio (HR) 3.53, 95% confidence interval (CI) HR 2.01–5.93; Figs. 1a and 2a). Furthermore, multivariate association evidenced a significant effect independently of patient sex, age, Ann Arbor stage (I-IV) and COO classification (*p*-value 2.06 × 10–9, HR 6.93, 95% CI HR 3.68–13.06). Cluster prediction on the independent test set classified a group of 20.31% of the patients in this cluster, and multivariate cox regression confirmed a significant and independent association with adverse outcome (*p*-value 5.43 × 10^− 3^, HR 6.80, 95% CI HR 1.76–26.26, Figs. 1b and 2b). Patient characteristics for botch clusters in the two cohorts can be consulted in Table 2.

**Table 2:** Patient characteristics by subgroups using 4-gene based clusterization

Cohort		GSE10846		GSE23501	
**Cluster**		**Cluster 1**	**Cluster 2**	**Cluster 1**	**Cluster 2**
**N. of cases**		184	49	51	13
**Sex (% male)**		60.32	46.94	74.51	61.53
**Median Age**		61	63	62	71
**COO**	**GCB**	41.30%	63.26%	27.45	38.46
	**ABC**	42.93%	28.57%	56.86	61.54
	**NC**	15.76%	8.16%	15.69	0

### Survival Prediction Using Random Forests

Clinical and molecular biology parameters were used to predict survival using random forests survival models. Initially, we tested the accuracy of the model using clinical data (patient sex, age and Ann Arbor stage), rendering C-indexes of 0.6340 and 0.6202 in the training and test cohorts, respectively (Table 3). Adding COO classification to the model improved concordance moderately (training c-index = 0.6761, test c-index = 0.6837). Notably, the inclusion of the previously described 4-gene expression-based clusterization increased discrimination capacity furhter (training c-index, 0.7059; test c-index, 0.7221).

**Table 3:** Random Forest models for overall survival prediction. C-index results are presented for each combination of variables in the training and test cohorts

	Training Cohort	Test Cohort
**GEP_0.01**	0.5934	0.6301
**GEP_0.05**	0.7530	0.6649
**GEP_0.1**	0.7783	0.7415
**Age, Gender, Stage**	0.6340	0.6202
**Age, Gender, Stage, COO**	0.6761	0.6837
**Age, Gender, Stage, 4-gene expression cluster**	0.6725	0.6971
**Age, Gender, Stage, COO, 4-gene expression cluster**	0.7059	0.7221
**GEP_0.1, 4-gene expression cluster**	0.7792	0.7558
**GEP_0.1, COO**	0.7784	0.7487
**Age, Gender, Stage, GEP_0.1**	0.7788	0.7522
**Age, Gender, Stage, GEP_0.1, 4-gene expression cluster**	0.7889	0.7416
**Age, Gender, Stage, GEP_0.1, COO**	0.7854	0.7538
**Age, Gender, Stage, COO, GEP_0.1, 4-gene expression cluster**	0.7896	0.7596
**Age, Gender, Stage, COO, GEP_0.1, 4-gene expression cluster** **(parameter optimized)**	0.8051	0.7615
**Age, Stage, COO, 4-gene expression cluster, 50 genes** **(variable reduction, parameter optimization)**	0.8404	0.7942

Afterwards, we studied survival predictability using expression data of those genes associated with overall survival (Supplementary Table 1). We initially analyzed different sets of genes in order to select the best combination. Survival prediction with those genes associated with survival at 3 different significance thresholds were selected: univariate cox q-value below 0.01 (GEP_0.01), 0.05 (GEP_0.05) and 0.1 (GEP_0.1), respectively. GEP_0.01 (3 genes) performed poorly (training c-index = 0.5934, test c-index = 0.6301). GEP_0.05 (12 genes) improved predictability (training c-index 0.7530, test c-index 0.6649). Notwhistandintly, the best prediction accuracy was achieved using GEP_0.1 (102 genes, Supplementary Table 2). This model achieved a high concordance with survival in the bootstrapped training cohort (c-index 0.7783) and in the test cohort (0.7415). Interestingly, only 6 of the genes included in this pattern match those of the Nanostring COO assay [22].

**Fig. 3:**
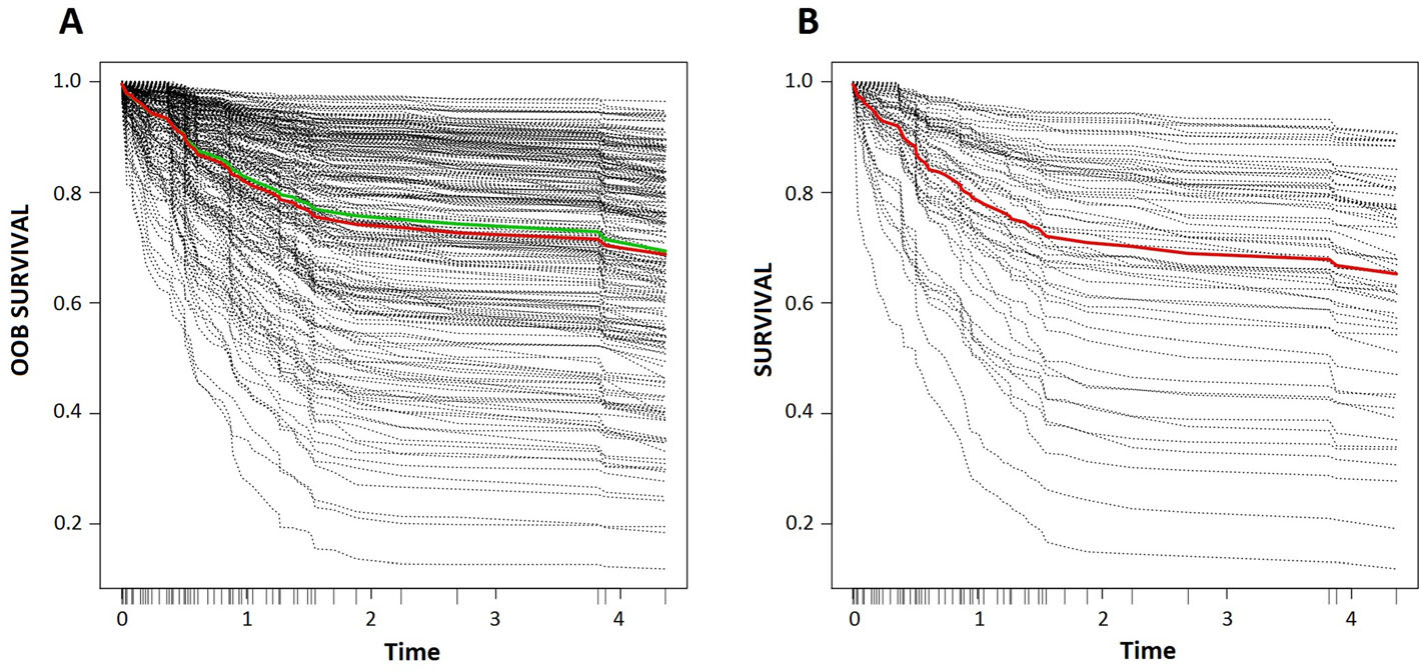
Predicted individual survival curves according to the most accurate random forest model (see text). **a**) Out-of-bag survival curves predicted for patients within the training cohort (discontinuous black lines). The thick red line represents overall ensemble survival and the thick green line indicates the Nelson-Aalen estimator. **b**) Individual survival curves predicted for patients within the test cohort (discontinuous black lines). The thick red line represents overall ensemble survival. Time scale is in years

Finally, we tested several combinations of GEP-based variables and clinical information (Table 3). The best model included clinical data, GEP_0.1, 4-gene expression clusterization and COO classification (c-indexes of 0.8051 and 0.7615 after parameter optimization in the training and test sets, respectively). By iteratively removing variables with negative or low importance values (< 1 × 10^− 4^) and tuning the “nsplit” parameter in the training cohort, an improved model was constructed based on 54 items (Supplementary Table 3), which achieved concordance indexes of 0.8404 in the training set and 0.7942 in the test set. Predicted individual survival curves according to this model for patients in both cohorts are represented in Fig. 3. Out-of-bag CRPS in the training set reached low values (∿0.1) even at 4 years of follow-up (Supplementary Fig. 1), and an stratified analysis by predicted mortality indicates a higher survival prediction accuracy for those patients with better prognosis. Notably, the importance of *MS4A4A* expression (probe id: 1555728_s_at) was the highest of all variables, followed by that of 4-gene expression clusterization. Furthermore, the expression of *SLIT2* (probe id: 230130_at), *NEAT1* (probe id: 220983_s_at), *CPT1A* (probe id: 203633_at), *IGSF9* (probe id: 229276_at) and *CD302* (probe id: 205668_at) were superior to that of COO classification.

## Discussion

In this study we present a new random forest model to predict survival in DLBCL based on clinical and gene expression data. Using cox regression and unsupervised clustering we identified a set of transcripts and a 4-gene expression cluster associated with overall survival. This information was used to fit predictive models of survival using random forests. The best model outperformed some of the most important prognostic factors known in the field of DLBCL. Moreover, its combination with clinical information and COO classification rendered survival predictions that show high concordance with reality.

The importance of gene expression biomarkers in DLBCL has been known for a long time. The COO classification was described almost two decades ago, linking DLBCL cellular ontogeny with clinical outcome [6]. Similarly, the prognostic role of double-expressor DLBCLs (DLBCLs with high expression of *MYC* and *BCL2* or *BCL6* but no accompained by their genomic rearrangement) was described several years ago [23]. Recent studies have reported interesting prognostic patterns using GEP in this field. For example, Ciavarella et al. [24] presented a new prognostic classification of DLBCL based on computational deconvolution of gene expression from whole-tissue biopsies, and detected transcriptomic prints corresponding to myofibroblasts, dendritic cells and CD4+ lymphocytes that were associated with improved survival [25]. Similarly, Ennishi et al. [26] used gene expression data to demonstrate the existence of a clinical and biological subgroup of GCB-DLCBLs that resemble double-hit lymphomas [24], whereas Sha et al*.* [27] identified a gene expression signature that characterizes a group of molecular high grade DLBCLs. Our results add to the growing evidence indicating that an improved transcriptome-based risk stratification beyond classical biomarkers is possible. Importantly, the 4-gene expression clusterization described here includes important driver genes of lymphomagenesis, such as *TNFRSF9* [26], *BIRC3* [28] and *BCL2L1* [29].

Other interesting studies have reported notable advances in DLBCL risk stratification. Reddy et al [30] used exome-sequencing data to create a genomic profile that improved state-of-the-art prognostic models. Nevertheless, their study was centered in prognostic groups rather than individualized predictions. In the same line, the accuracy of gene expression classifiers [24, 25, 27] for making personalized predictions was not tested. Recently, machine learning techniques were used by Biccler et al. [31] for individualized survival prediction in DLBCL. They reported a stacking approach that incorporated clinical and analytical variables in order to predict survival in DLBCL patients from Denmark and Sweden, achieving high performance (training cohort cross-validated c-index, 0.76; test cohort c-index, 0.74). In comparison, the results of our GEP-based random forest model suggest superior concordance indexes, and future head-to-head studies are needed to compare their predictive accuracies in an unbiased fashion. Surprisingly, we observed that transcriptomic data alone outperforms the combination of COO classification and limited clinical data. Another advantage of random forests is the quantification of variable importance. In this case, it is notable that variable importance for 6 individual transcripts was superior to that of COO classification.

This is the first approach to our knowledge that combines GEP with artificial intelligence for survival prediction of DLBCL patients. Machine learning models come along with substantial benefits in the area of survival prediction. Firstly, there is no prior assumption about data distribution, and complex interactions between the variables can be modelled. Secondly, they do not simply rely on pre-defined assumptions about the pathology (for example, COO status). Finally, gathered information is used to directly predict patient outcome, and individualized survival curves are obtained. These personalized approaches overcome the imperfect patient subgrouping derived from classical studies, and thus they are more useful in clinical practice. Our results might be particularly useful in order to select high-risk patients for inclusion in clinical trials.

This study, like many others in the field of disease prognostication, has some limitations. Firstly, some important prognostic features were not available for this study, such as fragility scores, *International Prognostic Index* (IPI), NCCN-IPI and *“double-hit”* status. Although the IPI has proven to improve prognostic stratification of gene expression arrays [16], there is still room for improvement of its predictive accuracy. In this line, the suboptimal performance of IPI and NCCN-IPI must be highlighted (c-indexes of 0.66 and 0.68 for IPI and NCCN-IPI, respectively; Biccler et al. [31]). Furthermore, comorbidities and cause of death were not reported in any of the two studies. Finally, competing variables such as the type of salvage therapy and/or having undergone an autologous stem cell transplantation were unknown. Additionally, some heterogeneity related to the inclusion of different high grade lymphoma subtypes (for example, double and triple-hit lymphomas) and the variability of techniques for COO classification used should be considered as potential limitations. Therefore, it is tempting to speculate that the combination of GEP with improved histopathological and clinical profiles will provide even better predictive models of DLBCL survival.

## Conclusion

This study presents a machine learning-based model for survival prediction of DLBCL patients based on GEP data and clinical information. The results of our model are superior to those described with current risk stratification scores (IPI, NCCN-IPI, COO status), and head-to-head comparisons with other published machine learning approaches in the field of DLBCL are needed in order to compare their predictive utility. We believe that our results will pave the way towards the establishment of individualized survival predictions that will be useful in clinical practice and might prompt the development of novel first-line therapeutic interventions for selected patients.

## Supplementary information

**Additional file 1 **: **Supplementary Figure 1**. Representation of out-of-bag CRPS over time.The red line represents CRPS for the whole population (see main text). Additionally, stratified CRPS by quartiles of out-of-bag ensemble (predicted) mortality are provided. Vertical lines above the x axis represent death events.

**Additional file 2 **: **Supplementary Table 1**. List of the probes associated with overall survival using univariate cox regression. Only those probes with FDR < 0.1 are shown. **Supplementary Table 2**. Microarray probes included in the GEP_0.1 gene expression pattern. **Supplementary Table 3**. Importance of the different variables in the best random forest model after variable pruning.

## Data Availability

All data is available in the properly referenced data repositories.

## Abbreviations

CI: Confidence interval
COO: Cell of origin
CRPS: Continuous rank probability score
DLBCL: Diffuse large B cell lymphoma
GEP: Gene expression profiling
HR: Hazard ratio
IPI: International prognostic index
