# Supplementary figures and images for: Improved personalized survival prediction of patients with diffuse large B-cell Lymphoma using gene expression profiling
Source: BMC Cancer. 2020 Oct 21;20:1017. doi: 10.1186/s12885-020-07492-y (PMC7579992; doi:10.1186/s12885-020-07492-y)

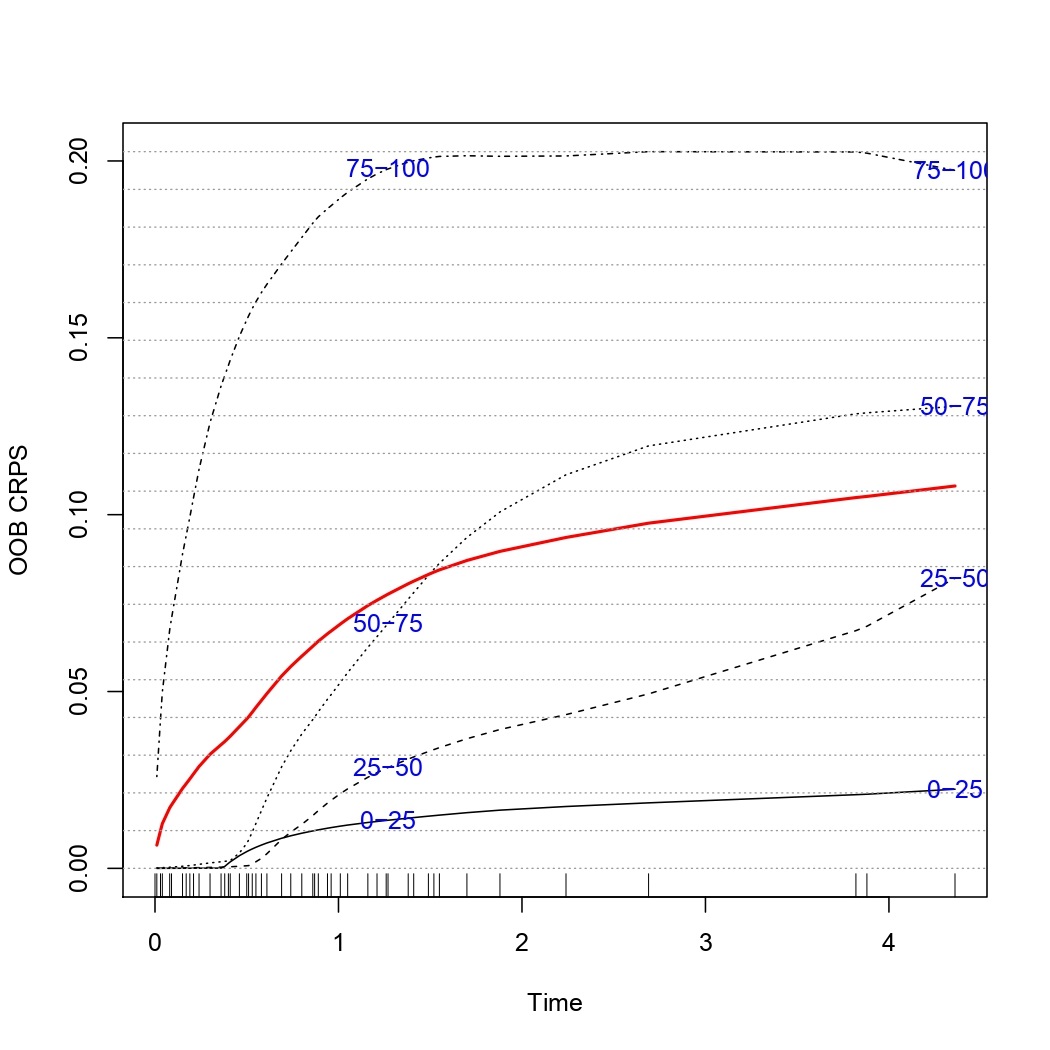

Supplement: Supplementary file 1 — Additional file 1 : Supplementary Figure 1. Representation of out-of-bag CRPS over time.The red line represents CRPS for the whole population (see main text). Additionally, stratified CRPS by quartiles of out-of-bag ensemble (predicted) mortality are provided. Vertical lines above the x axis represent death events. [file 12885_2020_7492_MOESM1_ESM.jpg]
